# Healthcare Provider Attitudes toward the Newly Developed COVID-19 Vaccine: Cross-Sectional Study

**DOI:** 10.3390/nursrep11010018

**Published:** 2021-03-23

**Authors:** Gasmelseed Ahmed, Zainab Almoosa, Dalia Mohamed, Janepple Rapal, Ofelia Minguez, Issam Abu Khurma, Ayman Alnems, Abbas Al Mutair

**Affiliations:** 1 Research Center, Almoosa Specialist Hospital, Al-ahsa 36342, Saudi Arabia; g.yousif@almoosahospital.com.sa (G.A.); z.almoosa@almoosahospital.com.sa (Z.A.); dalia@almoosahospital.com.sa (D.M.); janapple@almoosahospital.com.sa (J.R.); ofelia.minguez@almoosahospital.com.sa (O.M.); issam@almoosahospital.com.sa (I.A.K.); a.alnems@almoosahospital.com.sa (A.A.); 2 School of Mursing, Wollongong University, Wollongong, NSW 2522, Australia; 3 Nursing College, Princess Nora University, Riyadh 11564, Saudi Arabia

**Keywords:** healthcare providers, vaccination, Saudi Arabia, attitudes, acceptance, advocate, COVID-19

## Abstract

Background: During the long wait and the global anxiety for a vaccine against COVID-19, impressively high-safety and effective vaccines were invented by multiple pharmaceutical companies. Aim: We aimed to assess the attitudes of healthcare providers and evaluate their intention to advocate for the vaccine. Methods: This was a cross-sectional study conducted in a tertiary private hospital where an electronic survey was distributed among healthcare providers (HCPs). The survey contained two sections: socio-demographic characteristics and Likert-scale perception, with 72% internal consistency. Results: The response rate to the email survey was 37% (n = 236). In addition, 169 (71.6%) of respondents were women, with more than half (134, 56.8%) aged ≤35 years. A total of 110 (46.6%) had over 10 years of experience, and most of them were nurses (146, 62%). Univariate analysis revealed that older participants significantly accepted and advocated for the new vaccine more than the younger ones. In the multivariate analysis, men were significantly more likely than women to accept and advocate for the new vaccine, as were those with chronic illnesses. Participants with allergy were significantly less likely to accept the vaccine than others. odds ratio (OR) and *p*-values were 2.5, 0.003; 2.3, 0.04; and 0.4, 0.01, respectively. Conclusion: The acceptance rate for the newly-developed COVID-19 vaccines was average among HCPs. Sex, age, presence of chronic illnesses, and allergy were significant predictors of accepting the vaccine.

## 1. Background

No specific treatment was available for SARS-CoV-2; therefore, the rapid development of effective vaccines was urgently needed [1,2]. Many patients all over the world used human drugs off-label such as chloroquine, hydroxychloroquine, azithromycin lopinavir-ritonavir, favipiravir, remdesivir, ribavirin, interferon, convalescent plasma, hormones, and anti-IL-6 inhibitors based on either their in vitro antiviral or anti-inflammatory properties [3].

With multiple clinical vaccine studies ongoing, the target time for public distribution of a safe and efficient vaccine was projected as 18 months [4]. Therefore, to avoid the spread of COVID-19, measures to increase the acceptance of COVID-19 vaccines are critical. However, immunization program success depends on high vaccine acceptance versus rejection rates by healthcare providers, who play a crucial role in vaccination [5]. There is a demand to identify factors that may contribute to the acceptance and rejection of the newly-developed COVID-19 vaccine, especially doctors and nurses, who are known for being advocates of patients. Vaccine hesitancy is a global threat, so scientists must focus on understanding the underlying causes of this hesitancy to fight against vaccine misinformation [6]. Vaccine hesitancy is defined by its determinants; confidence, complacency and convenience are on the rise. Exploring the population’s concerns through research at the individual and community levels is the best practice to address the trust component of vaccine hesitancy and to promote vaccine acceptance [7] by effectively presenting science-based information, and accordingly presenting immunization as a social norm both in educational materials and in conversations or resilience. However, immunization trust-building and maintaining at the public level will take time [7].

Although immunization has successfully reduced the global burden of illness and deaths, the overall confidence in vaccines among communities can be affected by various concerns. Consequently, vaccine hesitancy can lead to vaccine refusal. Nowadays and due to the massive availability and accessibility of different modes communication media, the intensity, spread, and effects of public opinion on vaccines are speeding up information sharing, contributing to vaccine hesitancy and refusal [8].

While most of the world’s countries were in lockdown to limit the spread of COVID-19, scientists from all over the world were racing to provide proven treatment or develop vaccines against COVID-19. Their global access was a priority to end the pandemic [9]. The long-term solution to the COVID-19 pandemic will be a globally implemented and safe vaccination program, which will have both broad clinical and socioeconomic benefits. The vaccine must be delivered to the public as early as it is available to reduce morbidity and mortality from the COVID-19 pandemic. The vaccine must also be accepted by the public as well the healthcare community. Considering vaccine hesitancy as a major barrier to vaccine uptake, a high vaccine refusal rate could significantly affect the preventive goals [10].

In this study, we aimed to assess the attitudes of healthcare providers (HCPs) in a tertiary private hospital toward their acceptance and intention to advocate for the newly developed COVID-19 vaccine amongst patients, friends, and families. The study will identify the possible reasons behind HCP acceptance and rejection of the newly developed vaccines.

## 2. Methods

A cross-sectional study was conducted using a questionnaire. The study was conducted in a tertiary private hospital in the east region of Saudi Arabia, where an electronic survey was distributed to all HCPs. All respondent employees were enrolled consecutively in the study. An ethical approval to conduct the study was obtained from Almoosa Specialist Hospital’s Institutional Review Board (IRB) (log No: ARC-20.10.3).

The questionnaire consisted of two sections: the first was socio-demographic characteristics, gathering data on sex, age, educational status, years of experience, nationality, occupation, and marital status; the second used a Likert scale to gather information on perceptions (consisted of twelve items, including medical knowledge, trust in media, trust in manufacturers, and trust in policymakers and leaders). The validation test of the data collection instrument revealed an internal consistency of 72%.

A pre-defined justified sample size for our study was determined in reference to an effect size from similar study by Wang et al., reported 91.3% of participants stated that they would accept COVID-19 vaccination after the vaccine becomes available. Applying the standard categorical variable data sample equation [(n = z^2^ × PQ/e^2^), where z is confidence level, p is the reported effect size and e is the margin of error], expecting a 99% confidence level and accepting a narrow margin of error around 0.05, the optimal sample size for this study was calculated as 196 participants [11].

Prior to data collection, IRB approval was sought from the Almoosa Specialist Hospital (ARC-20.10.3). The Almoosa Specialist Hospital is a 220-bed tertiary private care center and the largest in the Al-ahsa region, Saudi Arabia. The study design was cross-sectional, approached using a survey administered to the hospital’s employees. The target population for this study was all the people serving in this facility, which has a local catchment population of over two million with all medical specialties including: adult; pediatric; neonatal; cardiology; oncology; internal medicine; infectious diseases; dermatology, gastroenterology; rheumatology; hematology; radiology; geriatrics; obstetrics and gynecology; neuroscience; nephrology; orthopedics; urology; surgery; ear, nose, and throat care; dental; burn; and intensive care.

## 3. Results

The response rate to the emailed survey was 37% (n = 236). In addition, 169 (71.6%) of respondents were women, with more than half (134, 56.8%) aged ≤35 years. A total of 110 (46.6%) had over 10 years of experience, and most of them were nurses (146, 62%) (Table 1). The common reasons for rejecting the vaccine are outlined in Table 2. Univariate analysis revealed that older respondents significantly accepted and advocated for the new vaccine more than the younger ones (53% vs. 47%, *p*-value = 0.003; Table 3). Multivariate analysis revealed that men were significantly more likely than women to accept and advocate for the new vaccine (OR = 2.5, *p*-value = 0.003), as were those who had chronic illnesses (OR = 2.3, *p*-value = 0.04). Participants with allergy were significantly less than others to accept the vaccine. The results also showed that the trust in healthcare providers is double the trust in other influential people (OR = 2.3, *p*-value = 0.05; Table 4). Healthcare providers’ specialties, graduation degree, and years of experience showed no statistically significance differences in the acceptance rate.

## 4. Discussion

In this study, we assessed the attitudes of healthcare providers toward the newly developed vaccine and evaluated their intention to advocate for it. The literature shows that several studies have been conducted on factors associated with the acceptance of vaccines among healthcare workers [12,13,14]. A cross-sectional study examining healthcare workers’ knowledge, attitude, and acceptance of influenza vaccination in Saudi Arabia has found that the acceptance and participation in influenza vaccination have markedly increased in the 2016 season compared with previous years, indicating highly motivated practitioners who seem prepared to encourage the adoption of influenza vaccination [15]. A systematic review focused on the factors influencing pandemic influenza vaccination among healthcare providers found that the H1N1 vaccine was likely to be accepted by healthcare workers if they perceived the vaccine as safe. Immunization effectively prevents infection of self and others and H1N1 is perceived as a serious and severe infection [16].

In the current study, we found above-average rates of acceptance and intention for advocating for the vaccine (56% and 60%, respectively). However, both accepting and advocating were reported by half of the study group (51.3%). The average rate of acceptance in our results is higher than for an online survey conducted in France in late March 2020 in a population aged 18 years: only 26% of participants agreed that they will use the vaccine against COVID-19 if it becomes available [17]. A survey conducted in 19 countries, which aimed to determine potential acceptance rates and factors influencing the acceptance of a COVID-19 vaccine, revealed differences in acceptance rates among participants ranging from almost 90% in China to less than 55% in Russia [18].

Despite the low rate of acceptance and the low trust caused by different factors, participants in this study mostly trust health policymakers and health leaders. Another study in India reported that vaccination decision-makers had different perceptions about building trust with the communities and foster engagement to function optimally toward achieving national vaccination goals [19].

A survey study on Israeli populations, which included both medical and non-medical staff, evaluated the current vaccination compliance rates and assessed whether participants would agree to receive a COVID-19 vaccine once available. The study results indicated that the rate of vaccine suspicion was high among medical professionals, which depended on the personal risk–benefit perception, which may be affected by misinformation about vaccine safety and efficacy. Due to the rapidly developed vaccine, many of the study respondents were non-compliant and raised fears about the safety of the vaccine. However, individuals who believe that they are at a higher risk of illness displayed greater vaccine acquiescence [20]. This finding agrees with our findings for our participants with chronic illnesses, where 22 of 32, (69%) of them reported to be willing to accept the newly developed vaccine. In a different study conducted in Hong Kong, a low rate of intention to accept COVID-19 vaccination and a high proportion of hesitation were found despite the evolution of the pandemic. As indicated by the authors, the reasons for this finding are related to suspicion regarding the safety and efficacy of the new vaccine [21].

Misinformation and conspiracy theories may decrease vaccine uptake, so the key to overcoming the anti-vaccination movement is establishing a consensus on how groups of the population will obtain access to the vaccine and mitigate any doubts and concerns that exist to generate demand for vaccinations. [22]. The threshold for COVID-19 herd immunity, as previously reported, was estimated to be between 55% and 82% of the total population. This could be significantly affected by a vaccine refusal rate of more than 10% to 15%, as reported in countries such as Australia [23]. An influential call to promote and advocate for the broader continuum of health and critical thinking is needed for preparing healthcare workers to meet the expected challenges of healthcare equity, environmental justice, and economic recovery [24]. Some health belief model (HBM) studies reported that a person will take health-related actions if they feel that a negative condition or side effects can be avoided, or if they have a positive expectation of taking a recommended action [25]. One of our concerns in the findings of our study is low level of acceptance and low trust in the newly produced vaccine. Similar findings were reported in study by Ozawa and Stack, revealing a wide vaccine confidence gap due to different factors, which necessitate building public trust by engaging all stakeholders including parents, healthcare providers, community leaders, policy makers, and the media [26].

## 5. Conclusions

The overall rate of acceptance for a newly developed COVID-19 vaccine among healthcare providers was average in this study. The results also demonstrated that sex, age, presence of chronic illnesses, and allergy are significant predictors for accepting the vaccine. It is strongly recommended that healthcare providers are prepared for a science- and evidence-based approach that addresses the safety and efficacy of the vaccines in the community to build and maintain public trust in the vaccine. Well-planned media and a positive influential campaign led by HCPs can be used to share transparent and scientific information with the community in terms of epidemiological details, scientific facts, and methodological process of the vaccine to promote critical thinking, which could result in increased confidence to optimize the uptake of the vaccine. Senior health are policy makers and leaders in public taking the vaccine will encourage more people to accept vaccination. The findings of the current study should be interpreted considering its several limitations: the cross-sectional approach of the study and using a survey tool with the lowest margin of internal consistency. Additionally, the survey was a self-administered questionnaire. Other limitations were the small sample taken from a single center, and the limited number of men and physicians participated in the survey. We acknowledge these limitations might potentially impact the study and limit generalizability of the findings. Hence, we recommend a future multicenter national study. Surveying a larger population by applying highly validated tool will rely on subjective rather than objective methods for increased understanding of valid perception and acceptance of the newly developed vaccine among healthcare providers.

## Figures and Tables

**Table 1:** Socio-demographic characteristics (n = 236).

Characteristics	N (%)
Sex	
Female	169 (71.6%)
Male	67 (28.4%)
Age group	
≤35 years	134 (56.8%)
>35 years	102 (43.3%)
Educational degree	
Graduate degrees (diploma and bachelor)	187 (79.2%)
Postgraduate degrees (master and Ph.D.)	49 (20.8%)
Country of origin	
Indian	75 (31.8%)
Philipino	73 (30.9%)
Saudi	41 (17.4%)
Others (different 11 countries ranging from 1 to 11 nurses)	47 (19.9%)
Do you have a comorbidity (any chronic disease)?	
Yes	032 (13.6%)
No	204 (86.4%)
Have allergy to medicine or food?	
Yes	35 (14.8%)
No	201 (85.2%)
Years of experience	
≤10	126 (53.4%)
>10	110 (46.6%)
Occupation	
Nurse	146 (61.9%)
Doctor	038 (16.1%)
Other	052 (22.0%)
Who do you trust the most for information on vaccination?	
Healthcare providers (HCPs)	190 (80.6%)
Leaders	14 (05.9%)
Media	6 (02.5%)
Policy makers	13 (05.5%)
None	13 (05.5%)
Accept the newly developed vaccine?	
Yes	131 (55.5%)
No	105 (44.5%)
Advocate for newly developed vaccine	
Yes	142 (60.1%)
No	094 (39.9%)
Both accept and advocate for newly developed vaccine	
Yes	121 (51.3%)
No	115 (48.7%)

**Table 2:** The common reasons for rejection of the vaccines (n = 236).

Characteristics	N (%)
Trust manufacturing country	
Agree	157 (66.5%)
Disagree	079 (33.5%)
I trust the manufacturing company of the vaccine	
Agree	159 (67.4%)
Disagree	077 (32.6%)
I believe vaccines are tested long enough for safety and efficacy	
Agree	150 (63.6%)
Disagree	086 (36.4%)
I think the media have created a negative impression about the vaccine	
Agree	091 (38.6%)
Disagree	145 (61.4%)
I think the vaccine’s industry is driven by financial motives	
Agree	136 (57.6%)
Disagree	100 (42.4%)
I believe forced vaccination by authorities provokes hesitancy	
Agree	172 (72.9%)
Disagree	064 (27.1%)

**Table 3:** Univariate analysis (n = 236).

Characteristics	Take and Advocate for Vaccine (%)	Will Not Advocate (%)	*p*-Value
Sex			
Female	70 (60.9%)	99 (81.8%)	
Male	45 (39.1%)	22 (18.2%)	0.0001
Age (years)			
≤35	54 (47.0%)	80 (66.1%)	
>36	61 (53.0%)	41 (33.9%)	0.003
Occupation			
Nurse	69 (60.5%)	77 (61.7%)	
Doctor	18 (15.4%)	20 (18.1%)	
All other HCPs	28 (24.1%)	24 (20.2%)	0.71
Degree of graduation			
Graduate degrees	89 (77.4%)	98 (81.0%)	
Postgraduate	26 (22.6%)	23 (19.0%)	0.49
Years of experience			
10 years or more	59 (51.3%)	51 (42.1%)	
Less than 10 years	56 (48.7%)	70 (57.9%)	0.16
Do you have any chronic disease?			
Yes	22 (19.1%)	10 (08.3%)	
No	94 (80.9%)	110 (91.7%)	0.02
Have allergy to medicine or food			
Yes	009 (07.8%)	26 (21.5%)	
No	106 (92.2%)	95 (78.5%)	0.003
Who do you trust the most for information on vaccination			
Health policymakers and leaders	18 (15.7%)	009 (07.4%)	
Others	97 (84.3%)	112 (92.6%)	0.048

**Table 4:** Multivariate analysis (n = 236). OR, odds ratio.

Characteristics	OR	95% CI	*p*-Value
Sex			
Male vs. female	2.5	(1.35–4.56)	0.003
Having allergy to medicine or food			
Those with allergy vs. others	0.4	(0.16–0.83)	0.02
Presence of chronic disease?			
Those with chronic disease vs. others	2.3	(1.02–5.39)	0.04
Who do you trust the most for information on vaccination			
Health policy makers and leaders vs. others	2.3	(0.99–5.37)	0.05

## Data Availability

Data used and analyzed in this study will be promptly available for the publisher upon request.
